# Spinal cord injury: is monitoring from the injury site the future?

**DOI:** 10.1186/s13054-016-1490-3

**Published:** 2016-10-05

**Authors:** Samira Saadoun, Marios C. Papadopoulos

**Affiliations:** Academic Neurosurgery Unit, St. George’s, University of London, Cranmer Terrace, Tooting, London SW17 0RE UK

**Keywords:** Blood pressure, CNS injury, Clinical trial, Microdialysis, Monitoring, Neurocritical care, Spinal cord injury, Surgery

## Abstract

This paper challenges the current management of acute traumatic spinal cord injury based on our experience with monitoring from the injury site in the neurointensive care unit. We argue that the concept of bony decompression is inadequate. The concept of optimum spinal cord perfusion pressure, which differs between patients, is introduced. Such variability suggests individualized patient treatment. Failing to optimize spinal cord perfusion limits the entry of systemically administered drugs into the injured cord. We conclude that monitoring from the injury site helps optimize management and should be subjected to a trial to determine whether it improves outcome.

## Background

Every year, 15–40 people per million suffer a traumatic spinal cord injury (TSCI) [1]. Many TSCI patients are initially admitted to a neurointensive care unit (NICU), but their management is variable. This review introduces novel concepts to aid the management of acute TSCI in NICU based on our findings that: 1) the dura causes spinal cord compression at the injury site; 2) each patient has an optimum spinal cord perfusion pressure; and 3) patient position in bed influences cord perfusion.

### Patient management

#### Surgical management

Some surgeons recommend early bony decompression, but others do not [2, 3]. The Surgical Timing in Acute Spinal Cord Injury Study showed better outcome at 6 months in patients who had decompressive surgery within 24 h compared with >24 h after cervical TSCI [4]. This study was underpowered, not randomized, and not blinded. In the UK [3] and internationally [2, 5] there is no consensus on the timing or even the role of surgery for TSCI. Below, we argue that early surgery is controversial because surgeons perform bony decompression, but fail to relieve the dural compression.

#### Medical and nursing management

There are no drugs that improve outcome after TSCI. The North American Spinal Cord Injury Studies suggested that methylprednisolone given within 8 h after TSCI improves outcome [6, 7], but their findings have been criticized; methylprednisolone is no longer standard of care [3, 8, 9]. The optimum mean arterial pressure (MAP) after TSCI is unknown. The American Association of Neurological Surgeons recommends a MAP of 85–90 mmHg for 7 days with little supporting evidence [10]. In the UK, blood pressure management is variable with 23 % of NICU specialists aiming for MAP >60 mmHg, 54 % >80 mmHg, and 15 % within 20 % of what is considered normal for age [3]. The effects on the injured cord of different anesthetics, altering arterial pCO_2_, administering vasopressors or mannitol, and patient position in bed are also unclear. In the UK, 96 % of neuroanesthesiologists avoid N_2_O [3] which elevates intracranial pressure (ICP), though its effect on intraspinal pressure (ISP) is unknown. Most surgeons fix the spine and mobilize patients early [2], but in the UK Midlands Spinal Injuries Centre patients were kept flat for 6 weeks after TSCI with an equally good outcomes [11]. Such variability in practice suggests that the optimum medical and nursing management are unknown. Below, we show how neuromonitoring may be used to optimize management.

### How to optimize management

#### History of monitoring from the injury site

In 2009, whilst studying the role of the water channel protein AQP4 in TSCI in mice, Dr Saadoun noticed that the injured cord was compressed against the dura, based on myelograms and ISP measurements [12]. She wondered whether the same occurs in TSCI patients. To investigate this, we set up a clinical trial termed Injured Spinal Cord Pressure Evaluation (ISCoPE), aiming to monitor ISP from the injury site in humans. By 2014, we had monitored the ISP in 18 patients with severe TSCI and had shown that ISP is high and potentially detrimental [13]. A detailed morphological and spectral analysis of the ISP signals followed [14]. Our studies led to the idea of dural spinal cord compression that causes compartmentalization at the injury site [15–17]. We thus evaluated the effect of duroplasty after TSCI [18]. In 2016, we reviewed all ISCoPE patients and demonstrated the safety and probe placement accuracy of the technique [19]. Multi-modality monitoring from the injury site was introduced in the same year [20], including a novel analysis technique using Kohonen self-organizing maps [21]. The ISCoPE studies are ongoing.

#### Brain *versus* spinal cord injury

Neuromonitoring is the standard of care for acute severe traumatic brain injury (TBI). We use the term neuromonitoring to mean monitoring ICP, tissue oxygenation, etc., in the NICU, rather than intraoperative neurophysiological monitoring. Though a recent study suggested that ICP monitoring does not improve outcome [22], the findings are confounded by a delay in the start of monitoring and no rehabilitation after discharge from the intensive care unit. Systematic reviews suggest that ICP monitoring in TBI reduces mortality [23] and increases the chance of a favorable outcome [24]. There are advocates of multi-modality monitoring from the injury site including microdialysis (MD), oxygen, blood flow, and electrocorticography based on the idea that secondary damage is multifactorial and arises not only from ischemia, but also from metabolic disturbance and spreading depolarizations. Probes are routinely introduced into the injured brain, but no such monitoring exists for TSCI.

Several reasons may account for the lack of neuromonitoring in TSCI. First, there is concern that inserting probes exacerbates spinal cord damage. Second, spinal probes require surgery for insertion. Third, many TSCIs are managed by orthopedic surgeons not accustomed to neuromonitoring. Fourth, the clinical impact of elevated ICP is easy to detect in anesthetized brain-injured patients as a loss of pupillary light reflex, dilated pupil, cardiovascular instability, and, ultimately, brain death. In contrast, increased ISP is difficult to detect in anesthetized TSCI patients. This requires monitoring of motor or sensory evoked responses, which is not routinely done. Fifth, CT scans are routinely used in TBI to decide management, e.g., decompressive craniectomy. In contrast, the injured cord is invisible on CT and, once the spine is fixed, it is difficult to see on MRI. For these reasons, ISP has not attracted the same interest as ICP.

#### Technique of monitoring from the injury site

**Fig. 1:**
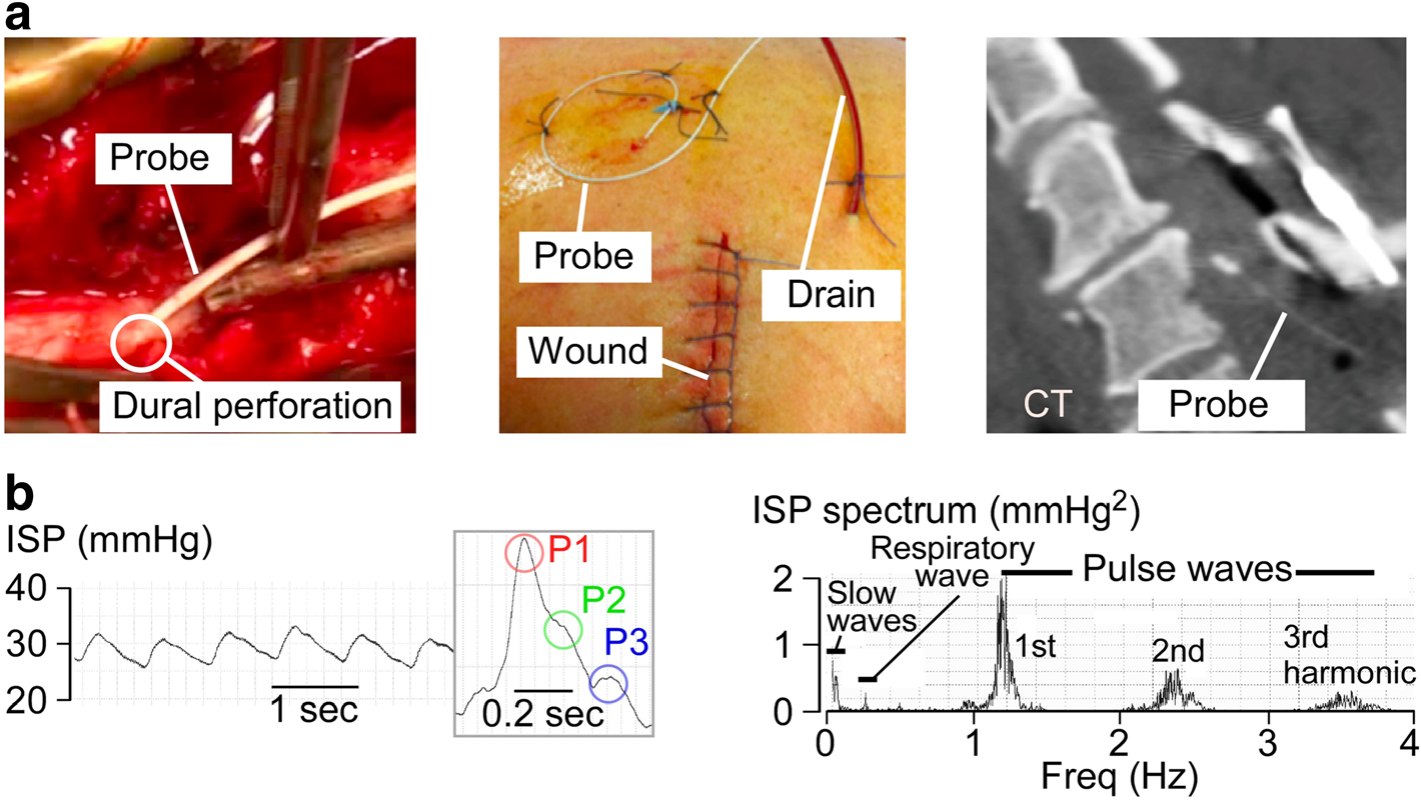
Intraspinal pressure (*ISP*) monitoring technique. **a** (*left*) Insertion of ISP probe through the dura. (*middle*) Surgical wound showing drain and ISP probe. (*right*) Postoperative computed tomography (*CT*) showing ISP probe. **b** (*left*) ISP signal. (*inset*) Magnified view of ISP waveform showing three peaks (P1, P2, P3). (*right*) Fourier transform of ISP signal. Modified from [13]

The probe is inserted intradurally during surgery at the injury site to monitor ISP for a week (Fig. 1a). The probe cannot be inserted in the NICU, thus precluding monitoring non-surgical patients. ISP monitoring is technically simple and safe based on data from 42 patients [19]. The probe is easily removed in the NICU.

The ISP and ICP waveforms are similar with three characteristic peaks, identical Fourier transforms, and identical shape change as ISP/ICP rise [14] (Fig. 1b). Thus, mathematically, the ISP signal can be subjected to the same analyses as the ICP signal [25]. Several parameters can be computed from these signals that provide physiological information about the injured cord (Table 1). It is important to note that we monitor ISP from the subdural space, which is less invasive than intraparenchymal ISP. When the injured cord is swollen and compressed against the dura, subdural ISP equals intraparenchymal ISP at the injury site [13, 17, 26]. After a mild TSCI, the spinal cord may not be compressed against the dura; in this case, the relation between subdural versus intraparenchymal ISP is unknown. To date, we have only monitored ISP from patients with severe TSCI.

**Table 1:** Comparison of brain and spinal cord physiological parameters

Brain parameter	Spinal cord parameter	Similarities	Differences	Reference
Intracranial pressure (ICP)	Intraspinal pressure (ISP)	ICP and ISP waveforms similar with same three peaks and similar Fourier transforms	Injury site ISP > ISP above or below, but ICP similar throughout. ICP but not ISP reduced with mannitol or hyperventilation.	[13, 17, 35, 44]
Cerebral perfusion pressure (CPP) = MAP – ICP	Spinal cord perfusion pressure (SCPP) = MAP – ISP	CPP and SCPP waveforms similar. CPP and SCPP can be increased with vasopressors	SCPP at injury site differs from SCPP above or below, but CPP similar in brain	[13, 17, 35, 44]
Optimum cerebral perfusion pressure (CPP_opt_)	Optimum spinal cord perfusion pressure (SCPP_opt_)	U-shape PRx vs CPP, sPRx vs SCPP. Minimum is CPP_opt_ or SCPP_opt_. CPP_opt_ and SCPP_opt_ individualized	Overall CPP_opt_ ~75 mmHg whereas overall SCPP_opt_ ~90 mmHg	[13, 14, 17, 35, 44]
Pressure reactivity index (PRx)	Spinal Pressure reactivity index (sPRx)	Running correlation between MAP and ICP/ISP	PRx is global, but sPRx is for injury site	[13, 14, 35, 44]
Compensatory volume reserve (RAP)	Spinal compensatory volume reserve (sRAP)	Running correlation between mean ICP/ISP and ICP/ISP pulse amplitude	RAP is global, but sRAP is for injury site	[13, 14, 35, 44]

It is also possible to perform multi-modality monitoring by placing ISP and MD probes on the spinal cord surface at the injury site [20]. In our recent study of 14 TSCI patients, we found that surface MD monitoring is safe [20]. There was severe metabolic derangement at the injury site that correlated with the severity of injury. Below, we discuss how MD can be used to determine the optimum spinal cord perfusion pressure (SCPP_opt_) and optimum tissue glucose concentration as well as maximize the penetration of systemically administered drugs into the injury site.

#### Animal models

After TSCI in mice, the injured cord swells and is compressed against the dura thus generating high ISP [12]. Mice that lack the water channel protein aquaporin-4 have reduced spinal cord edema after TSCI with improved outcome. Reducing the elevated ISP in a rat contusion TSCI model also improved outcome [27]. In a pig contusion TSCI model, increased thecal sac dimensions were associated with reduced cord compression and reduced injury site ISP [28]. Durotomy reduced intraparenchymal ISP after TSCI in ex-vivo pig spinal cords [29]. In a rat contusion TSCI model, durotomy with duroplasty improved outcome more than durotomy without duroplasty [27]. After rat TSCI, durotomy without duroplasty was associated with more macrophage accumulation, cystic cavitation, and fibroblast proliferation than durotomy with duroplasty [30]. Dural continuity also prevented epidural and spinal cord fibroblast proliferation and scar formation. Together, the animal experiments suggest that opening the dura is advantageous by relieving ISP, but a dural patch is required to reduce spinal cord inflammation and scarring. We have recently completed a phase II trial of expansion duroplasty for TSCI, described below.

### Novel clinical concepts

#### Intraspinal pressure and spinal cord perfusion pressure

Low MAP is detrimental by causing ischemic cell death. Increasing MAP is, therefore, likely to be beneficial. Even if the injury site is not viable, increasing MAP may still be beneficial by preventing upward extension of the injury, based on the idea of a critically ischemic penumbra. Recent studies suggest that the duration of hypotension after acute TSCI, defined as MAP <85 mmHg, correlates with worse outcome [31], though use of vasopressors to treat hypotension causes complications [32] especially in older TSCI patients [33]. In our view, what actually matters is not MAP, but MAP minus ISP, i.e., spinal cord perfusion pressure (SCPP). Because of the lack of ISP monitoring to date, the concept of SCPP has not been developed, but is analogous to cerebral perfusion pressure (CPP) for TBI. To re-phrase the first statement, low SCPP is probably detrimental.

**Fig. 2:**
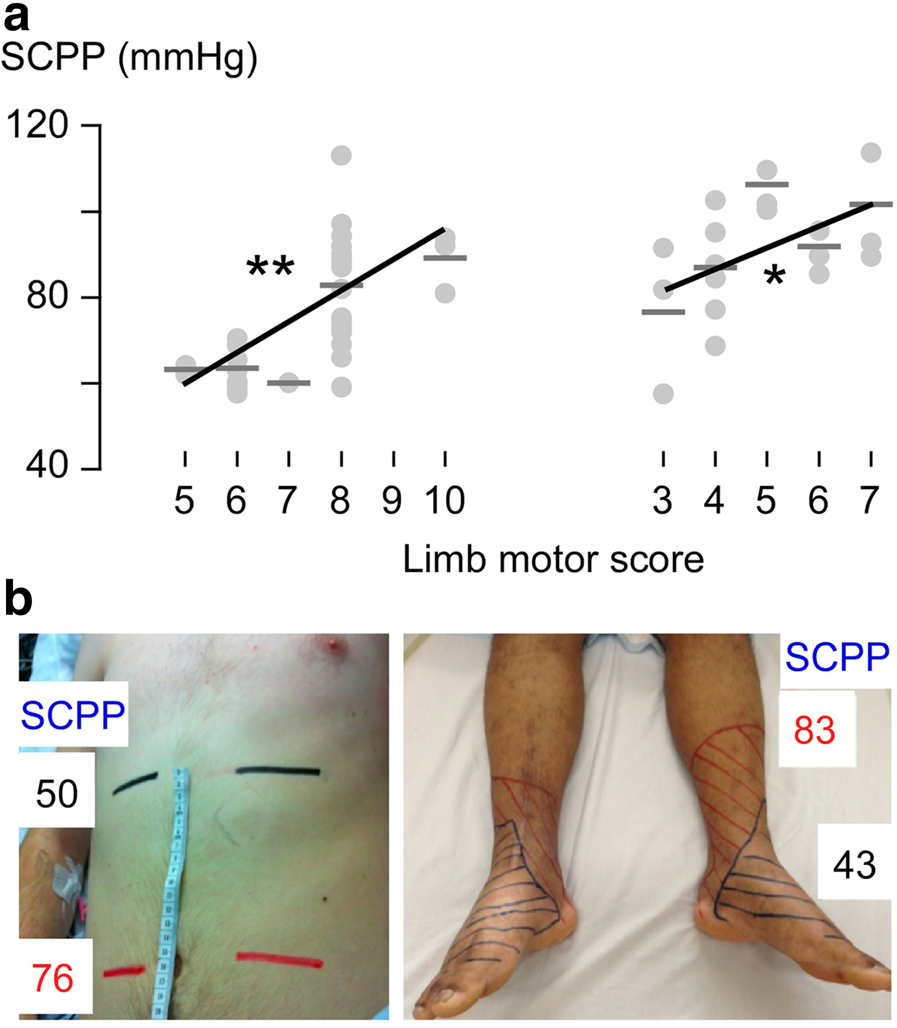
Spinal cord perfusion pressure (SCPP) correlates with outcome. **a** Plot of total limb neurological score versus SCPP for two AIS grade C TSCI patients. **b** Change in sensory level to pinprick in response to change in SCPP. Modified from [13]

We have several lines of evidence that this is so. First is the analogy between TBI and TSCI. After TSCI, SCPP is low (typically <60 mmHg) [13]; TBI patients with CPP <60 mmHg are at high risk of brain death [34]. Second, increasing SCPP increases the limb motor score in some AIS (American spinal injuries association Impairment Scale) grade C patients [13] and improves the sensory level in some AIS grade A patients (Fig. 2). Third, increasing SCPP increases the amplitude of motor evoked potentials recorded from below the injury in AIS grade C patients or just above the injury in AIS grade A patients [13]. Fourth, increasing SCPP improves spinal cord autoregulation [13, 14]. Fifth, increasing SCPP is associated with increased nutrient supply (tissue glucose), reduced cell membrane lysis (glycerol), excitotoxicity (glutamate), and tissue ischemia (lactate-to-pyruvate and lactate-to-glucose ratios). Sixth, increasing SCPP is associated with increased blood flow to the injury site [13]. Together, these data suggest that the injured spinal cord is ischemic and that increasing SCPP reduces ischemia. Below we show that too high a SCPP may be detrimental.

There are clinically important differences between ICP versus ISP and CPP versus SCPP (Table 1) [13]. Increasing the dose of sevoflurane from 1.0 to 1.5 minimum arteriolar concentration causes ICP to rise, but does not affect ISP. Reducing p_a_CO_2_ by 1 kPa, which reduce ICP by vasoconstriction, does not influence ISP. Administering an intravenous 100 mL bolus of 20 % mannitol reduces ICP, but not ISP. The only medical maneuver that increases SCPP is increased dose of vasopressors. Therefore, the therapeutic options to improve perfusion at the injury site are limited for TSCI compared with TBI.

#### Pressure reactivity and compensatory reserve

Pressure reactivity is the ability of smooth muscle in the walls of arteries and arterioles to respond to changes in arterial pressure to keep blood flow constant. We define spinal pressure reactivity index (sPRx) as the running correlation coefficient between ISP and MAP. Therefore, ^–^1 ≤ sPRx ≤ ^+^1. When sPRx ≤0, spinal cord pressure reactivity is intact and when sPRx >0, pressure reactivity is impaired. sPRx for TSCI is analogous to the pressure reactivity index (PRx) for TBI [25, 35].

The Monro-Kellie doctrine states that inside the adult skull there is blood, cerebrospinal fluid (CSF), and brain tissue in a state of volume equilibrium; an increase in volume of one component is compensated by an equivalent decrease in volume of another. A small increase in brain volume does not lead to increases in ICP because CSF and venous blood are displaced into the spinal canal. Once the ICP is ~25 mmHg, small increases in brain volume cause marked elevations in ICP. This is the basis of an exponential relationship between ICP and intracranial volume and the concept of RAP, which is a running correlation coefficient (R) between mean ICP amplitude (A) and ICP pulse amplitude (P). RAP ~0 indicates good compensatory reserve, whereas RAP ~1 indicates that ICP rises greatly with a small increase in volume [25]. Here, we argue that the Monro-Kellie doctrine may also apply to the injured cord. After severe TSCI, the pia is damaged, evidenced by the observations that the injured cord appears swollen on MRI [16] and, at the injury site, subdural ISP equals intraparenchymal ISP [17]. Spinal cord swelling may generate forces radially and rostro-caudally. Since most neuronal fibers run rostro-caudally, cord edema will produce radial rather than rostro-caudal cord expansion. The denticulate ligaments and nerve roots may also restrict rostro-caudal cord expansion. If rostro-caudal spinal cord expansion were possible, then the spinal cord above the injury would be displaced upward whereas the spinal cord below the injury would be displaced downward. Such displacements are not seen on MRI scans; instead, there is radial swelling of the injured cord against the dura. The observations that, after a laminectomy, the dural sac diameter at the injury site appears the same as above or below, and that ISP at the injury site remains high, suggest that the spinal dura is non-distensible. Thus, as the injured cord swells, the compensatory mechanisms of displacing CSF and venous blood become exhausted and ISP rises. This means that the injury site probably obeys the Monro-Kellie doctrine and the concept of compensatory volume reserve likely applies, quantified using spinal RAP (sRAP) by analogy with brain RAP [13, 14]. sRAP ~0 indicates good pressure-volume compensatory reserve and, when RAP ~1, the swollen spinal cord is on the steep part of the pressure-volume curve. We showed that as ISP rises >10 mmHg, sRAP increases, and as SCPP rises >40 mmHg, sRAP decreases [13, 14].

#### Overall optimum spinal cord perfusion pressure

If we continue to increase SCPP, there must be a point beyond which SCPP becomes detrimental. Mechanisms of over-perfusion injury include increased spinal cord edema and intraparenchymal hemorrhage. Since too low and too high values of SCPP are detrimental, there must exist an optimum spinal cord perfusion pressure (SCPP_opt_) in between.

**Fig. 3:**
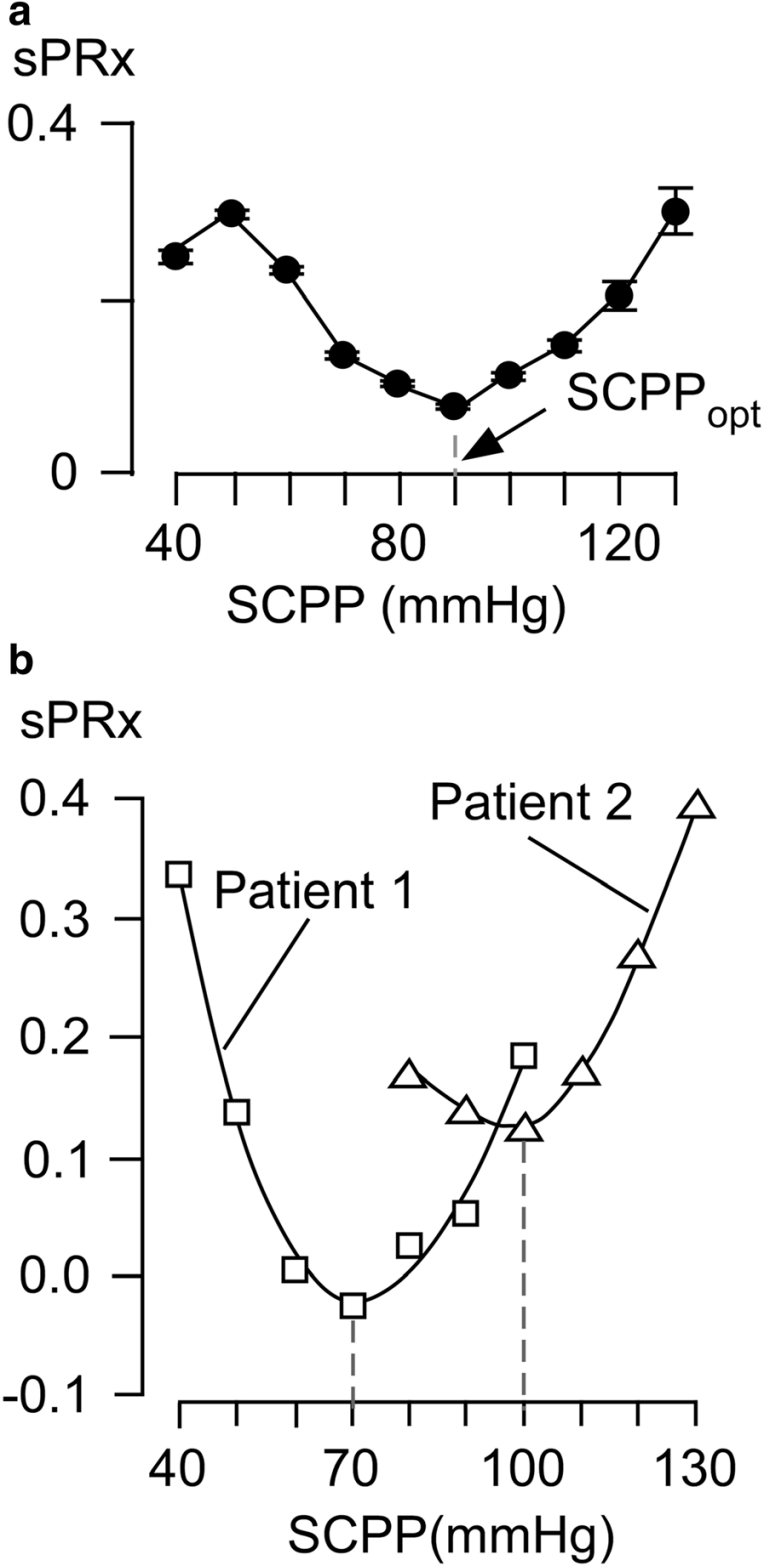
Spinal pressure reactivity index (*sPRx*) and optimum spinal cord perfusion pressure (*SCPP*_*opt*_). **a** Plot of sPRx versus SCPP for 18 patients. **b** Individual plots of sPRx versus SCPP for two patients. The minimum value corresponds to SCPP_opt_. Adapted from [13] and [18]

We define SCPP_opt_ as the perfusion pressure that minimizes sPRx. A plot of the sPRx against SCPP gives a U-shaped relationship, which supports the notion that not only hypo-perfusion, but also hyper-prefusion is detrimental [25, 35]. The minimum of the sPRx versus SCPP curve is the SCPP at which the injury site autoregulates best, i.e., SCPP_opt_. We showed that SCPP_opt_ is approximately 90 mmHg (Fig. 3a) [13, 18]. Autoregulation is abnormal even at SCPP_opt_ because sPRx >0. A U-shaped relationship also exists between PRx and CPP in severe TBI. The brain is more extensively vascularized than the spinal cord, which may explain why, in general, optimum cerebral perfusion pressure (CPP_opt_) is less than SCPP_opt_ at 70–75 mmHg. Compared with TBI patients managed at CPP near CPP_opt_, those managed below CPP_opt_ have higher mortality whereas those managed above CPP_opt_ have more severe disabilities [36]. The effect on neurological outcome of managing TSCI patients above or below SCPP_opt_ is unknown. SCPP_opt_ can also be defined as the SCPP that optimizes injury site metabolism (i.e., highest glucose, lowest glutamate, lowest glycerol, lowest lactate-to-pyruvate and lactate-to-glucose ratios). MD data after TSCI suggest that SCPP_opt_ = 90–100 mmHg [20], similar to the SCPP_opt_ determined from sPRx.

Let us put this SCPP_opt_ in context. After severe TSCI, ISP is typically 20–40 mmHg. Therefore, to achieve SCPP of ~90 mmHg, MAP has to be 110–130 mmHg, which is substantially higher than the MAP of 85–90 mmHg recommended by the American Association of Neurological Surgeons [10]. Most patients have a cervical injury and are, therefore, hypotensive from damage to the sympathetic pathways. To maintain MAP 110–130 mmHg, high-dose vasopressors are required, which are associated with drug-related complications [32]. One way to reduce the dose of vasopressors is by surgical decompression, discussed in detail below (‘Dural spinal cord compression’).

#### Patient-specific optimum spinal cord perfusion pressure

Figure 3a shows sPRx against SCPP by combining data from several patients. Most patients have their own U-shaped curve and individualistic SCPP_opt_ (Fig. 3b). Factors that may contribute to the variability of SCPP_opt_ between patients include the extent of microvascular damage, pre-existing hypertension, mechanism of injury, and genotype. The spinal cord metabolic response to injury is also patient-specific [21]. The concept that SCPP_opt_ and injury site metabolism vary between patients suggests that universal management protocols are insufficient and that neuromonitoring is essential to achieve individualized management.

#### Injury site compartmentalization

**Fig. 4:**
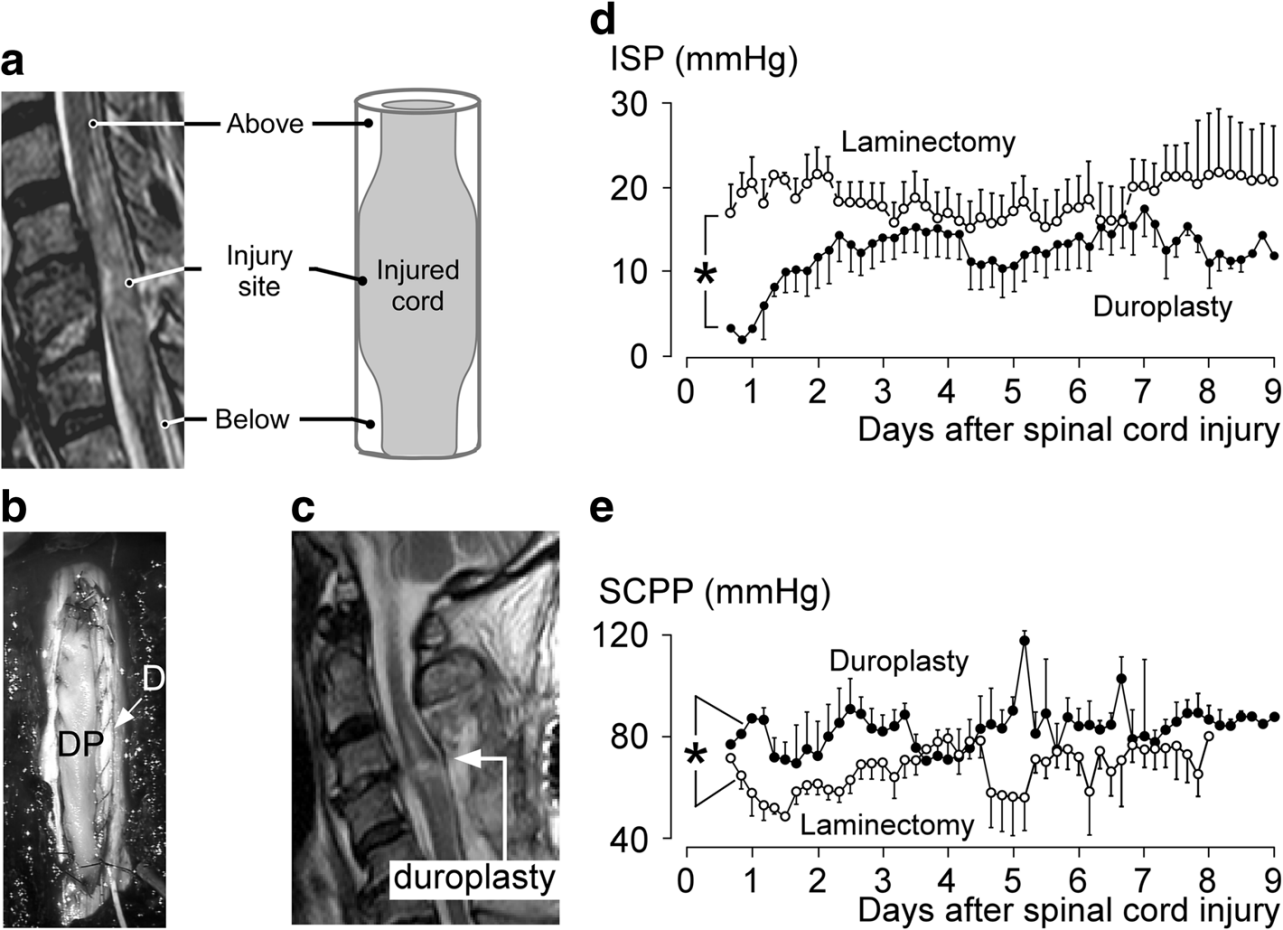
Expansion duroplasty. **a** Three intradural compartments form after TSCI—above, at, and below the injury site. **b** Intraoperative photo showing duroplasty. *D* dura, *DP* dural patch. **c** Postoperative MRI showing the swollen, injured cord herniating into the duroplasty. **d** Intraspinal pressure (*ISP*) and **e** spinal cord perfusion pressure (*SCPP*) versus days after injury for 10 patients who had laminectomy and 11 patients who had laminectomy + duroplasty. Mean ± standard error. *P < 0.05. Adapted from [13] and [18]

By monitoring two pressures simultaneously [13], or ISP along the injured cord [17], we showed that three intradural compartments form after severe TSCI: 1) above the injury; 2) at the injury; and 3) below the injury [15] (Fig. 4a). These compartments do not communicate between themselves; therefore, each compartment has a different pressure. The highest ISP is at the injury site where the swollen, injured cord is compressed against the dura. Because of compartmentalization, monitoring CSF pressure from below the injury does not provide information about ISP at the injury site. Draining CSF with a lumbar catheter has been proposed as a treatment for TSCI [37]. CSF drainage is unlikely to improve SCPP in TSCI because, at the injury site, there is no CSF around the spinal cord. In 65 patients with TSCI, 63 (97 %) had cord compression on MRI; compression was extradural in 75 % and dural in 25 % [16]. After bony decompression, most patients still had high ISP [13, 18], suggesting dural cord compression in most. Two other lines of evidence support the lack of communication between the CSF compartments above and below the injury. After TSCI, little CSF can be drained from the lumbar region and, when the lumbar drain is transduced, there is a non-pulsatile signal [37]. Draining CSF through a lumbar catheter is also potentially detrimental by causing downward herniation of the injured cord.

Compartmentalization may be a feature of the severity of TSCI and the surrounding CSF space; if TSCI is mild or the CSF space is large, the spinal cord will not be compressed by the dura and, therefore, the three compartments will not form. It is unknown if, in the first few days after TSCI, compartmentalization is constant or dynamic or if compartmentalization is more common in some TSCI types, e.g., flexion/distraction.

#### Dural spinal cord compression

Why is bony decompression for TSCI controversial? The answer may be the dura surrounding the damaged cord, which is poorly distensible. Even if early bony decompression is performed, the swollen cord remains compressed against the dura. Dural spinal cord compression explains why ISP at the injury site remains high even after realignment of the spinal fracture, fixation, and laminectomy [13, 18]. The concept of dural compression is well established for TBI, where decompressive craniectomy involves not only removing bone but also opening the dura.

The idea of dural compression is not appreciated after TSCI, though compression of the swollen, injured spinal cord against the surrounding dura is evident on postoperative MR scans [13, 16, 18] (Fig. 4a). The term ‘spinal decompression’ refers to removing extradural compression such as bone fragments *without* opening the dura, whereas the term ‘brain decompression’ refers to removing bone *and* opening the dura. Surgeons developed the concept of spinal decompression to treat degenerative spinal diseases such as canal stenosis where neural tissue is compressed from outside the dura. Unlike degenerative spinal pathologies, however, in TSCI the damaged spinal cord itself is swollen against the surrounding dura and, therefore, dural spinal cord decompression is essential. *We, therefore, propose that the concept of decompression for TSCI be modified to include opening the dura*. Serial MR scans of TSCI patients indicate that dural spinal cord compression resolves with a half-life of 8.7 days [16]. The slow rate of resolution of dural spinal cord compression supports early surgical decompression to reduce prolonged use of vasopressors.

We have recently conducted a phase II non-randomized trial comparing laminectomy versus laminectomy plus expansion duroplasty (Fig. 4b–e) [13, 16, 18]. After laminectomy plus expansion duroplasty, the injured cord herniates into the extra space that has been created, which suggests that the swollen cord was compressed by the dura. Laminectomy plus expansion duroplasty decompressed the injured cord more effectively than laminectomy without duroplasty, as evident on MRI. Laminectomy plus expansion duroplasty also reduced ISP, increased SCPP, and lowered the sPRx versus SCPP curve downward with the minimum at 90 mmHg. Duroplasty takes about 10–15 min to perform and is safe; the main side effect is asymptomatic pseudomeningocele in about half of patients that resolves within 6 months. This study was powered to detect an increase in dural diameter, spinal cord decompression by MRI, a reduction in ISP and sPRx, as well as an increase in SCPP, but was underpowered to detect neurological improvement.

#### Nursing care: lying supine may be detrimental

In patients who have had laminectomy, wound compression increases ISP and reduces SCPP due to transmission of the compressive forces to the cord [13]. The ISP rise was up to 20 mmHg, enough to aggravate cord damage. Lying supine is also associated with higher ISP, by 2–4 mmHg, compared with lying laterally [18]. This pressure difference was more marked for thoracic than cervical TSCI [19], probably because, when supine, the cervical cord is less compressed due to its lordosis whereas the kyphotic thoracic cord becomes more compressed. No difference in ISP or SCPP was observed in non-laminectomized patients. In one thoracic TSCI patient, the difference in ISP between supine and lateral position was 18 mmHg [19]. These findings suggest that, after TSCI, patients who had laminectomy should be nursed on their side to avoid aggravating cord damage by increasing ISP and reducing SCPP.

#### Neuroprotective drug trials

Issues to consider when designing drug trials for TSCI have been described elsewhere [38–41]. Patient age, site and severity of injury, and rehabilitation influence outcome. Such heterogeneity means that many patients are required. The formation of clinical networks may facilitate patient recruitment into trials, whereas MR imaging and electrophysiological outcome measures may reduce the number of patients required. Another issue is penetration of systemically administered drugs into the injury site, especially since patients with severe cervical TSCI are hypotensive with high ISP. Though the blood-spinal cord barrier opens after acute TSCI thus favoring drug entry into the injury site, we have recently shown that SCPP is also a major, but unappreciated, determinant of drug entry [20]. An increase in SCPP by 10 mmHg increases dexamethasone penetration into the injury site three-fold. Ongoing trials of minocycline and riluzole [42] as well as completed drug trials of methylprednisolone [6, 7] have not optimized SCPP. It is possible that drug trials for acute TSCI fail because of inadequate drug penetration at the injury site.

### Limitations

At present, our unit is the only one that performs neuromonitoring; it is vital that others reproduce our findings. With our technique, probes are inserted intraoperatively, which limits their use. In the future, the method should be refined to insert probes percutaneously in the NICU. This will allow neuromonitoring to start earlier and widen its use to non-TSCI, such as edematous transverse myelitis where not only inflammation but also ischemia may play a role [43]. Several of the concepts we introduced, such as SCPP_opt_, make physiological sense, but are presently theoretical. Evidence is required that SCPP_opt_ has clinical value, e.g., by showing that patients managed with SCPP close to SCPP_opt_ have better outcomes than those with large SCPP deviation from SCPP_opt_. Even if SCPP_opt_ is clinically important, it is computed after the monitoring has finished; a continuous SCPP_opt_ is required, which is displayed in real-time in the NICU. Another issue is timing of surgery, which is delayed in many units. It is, therefore, unknown if ultra-early bony decompression prevents cord swelling thus precluding the need for ISP monitoring or duroplasty. With further studies, these limitations can be overcome.

## Conclusions

We describe how monitoring from the injury site may guide the management of TSCI. Ultimately, a randomized controlled trial is required to determine if monitoring and SCPP optimization improve outcome, which can only be achieved by international collaboration.

## Abbreviations

AIS: American spinal injuries association Impairment Scale
CPP: Cerebral perfusion pressure
CPP_opt_: Optimum cerebral perfusion pressure
CSF: Cerebrospinal fluid
ICP: Intracranial pressure
ISCoPE: Injured Spinal Cord Pressure Evaluation
ISP: Intraspinal pressure
MAP: Mean arterial pressure
MD: Microdialysis
NICU: Neurointensive care unit
PRx: (brain) pressure reactivity index
RAP: (brain) compensatory volume reserve
SCPP: Spinal cord perfusion pressure
SCPP_opt_: Optimum spinal cord perfusion pressure
sPRx: Spinal pressure reactivity index
sRAP: Spinal compensatory volume reserve
TBI: Traumatic brain injury
TSCI: Traumatic spinal cord injury
